# The burden of *clostridium difficile* infection: estimates of the incidence of CDI from U.S. Administrative databases

**DOI:** 10.1186/s12879-016-1501-7

**Published:** 2016-04-22

**Authors:** Margaret A. Olsen, Yinong Young-Xu, Dustin Stwalley, Ciarán P. Kelly, Dale N. Gerding, Mohammed J. Saeed, Cedric Mahé, Erik R. Dubberke

**Affiliations:** Division of Infectious Diseases, Washington University School of Medicine, Campus Box 8051, 660 S. Euclid Ave, St. Louis, 63110 MO USA; Department of Surgery, Washington University School of Medicine, St. Louis, MO USA; Department of Psychiatry, Geisel School of Medicine at Dartmouth, Hanover, NH USA; Department of Medicine, Harvard Medical School, Boston, MA USA; Edward Hines Jr. Veterans Affairs Hospital, Hines, IL, USA and Loyola University Chicago Stritch School of Medicine, Maywood, IL USA; Sanofi-Pasteur, Lyon, France

**Keywords:** Clostridium difficile, Incidence, Administrative data, Claims data, Medicare data, Hospital-acquired infection

## Abstract

**Background:**

Many administrative data sources are available to study the epidemiology of infectious diseases, including *Clostridium difficile* infection (CDI), but few publications have compared CDI event rates across databases using similar methodology. We used comparable methods with multiple administrative databases to compare the incidence of CDI in older and younger persons in the United States.

**Methods:**

We performed a retrospective study using three longitudinal data sources (Medicare, OptumInsight LabRx, and Healthcare Cost and Utilization Project State Inpatient Database (SID)), and two hospital encounter-level data sources (Nationwide Inpatient Sample (NIS) and Premier Perspective database) to identify CDI in adults aged 18 and older with calculation of CDI incidence rates/100,000 person-years of observation (pyo) and CDI categorization (onset and association).

**Results:**

The incidence of CDI ranged from 66/100,000 in persons under 65 years (LabRx), 383/100,000 in elderly persons (SID), and 677/100,000 in elderly persons (Medicare). Ninety percent of CDI episodes in the LabRx population were characterized as community-onset compared to 41 % in the Medicare population. The majority of CDI episodes in the Medicare and LabRx databases were identified based on only a CDI diagnosis, whereas almost ¾ of encounters coded for CDI in the Premier hospital data were confirmed with a positive test result plus treatment with metronidazole or oral vancomycin. Using only the Medicare inpatient data to calculate encounter-level CDI events resulted in 553 CDI events/100,000 persons, virtually the same as the encounter proportion calculated using the NIS (544/100,000 persons).

**Conclusions:**

We found that the incidence of CDI was 35 % higher in the Medicare data and fewer episodes were attributed to hospital acquisition when all medical claims were used to identify CDI, compared to only inpatient data lacking information on diagnosis and treatment in the outpatient setting. The incidence of CDI was 10-fold lower and the proportion of community-onset CDI was much higher in the privately insured younger LabRx population compared to the elderly Medicare population. The methods we developed to identify incident CDI can be used by other investigators to study the incidence of other infectious diseases and adverse events using large generalizable administrative datasets.

**Electronic supplementary material:**

The online version of this article (doi:10.1186/s12879-016-1501-7) contains supplementary material, which is available to authorized users.

## Background

*Clostridium difficile* infection (CDI) incidence in the United States has increased dramatically since 2000 [1, 2]. The number of discharges from non-federal, acute care hospitals assigned the International Classification of Diseases, Ninth Revision, Clinical Modification (ICD-9-CM) diagnosis code for CDI (008.45) increased by 2.7-fold between 2000 and 2012 using data from the Healthcare Cost and Utilization Project (HCUP) Nationwide Inpatient Sample (NIS) [3]. CDI was estimated to cause as many as 14,000 deaths in 2007 and an attributable mortality ranging from 5.7 % in endemic settings to 16.7 % in severe outbreaks since 2000 [2, 4–7].

Much research has focused on identifying specific risk factors for CDI, but this might not be the best approach to identify high risk populations. The results of risk factor studies have not always been consistent [8–15], with potential reasons for discrepancies including differences in patient populations, data availability, and/or study definitions. These differences limit both the ability to compare results across studies and the generalizability of results, making it difficult to identify which populations have the highest CDI burden and how to best target CDI prevention practices.

Billing and claims data (referred to collectively as administrative data) are increasingly used for health services and outcomes research because of large population sizes, generalizability of findings, and the ability to follow individuals across the spectrum of health care. Unfortunately there is no single, comprehensive database in the U.S. that can be used to identify all populations at risk for CDI. In order to better understand the epidemiology of CDI we applied common definitions to identify and classify CDI from five large administrative databases, the Medicare 5 % Sample, HCUP State Inpatient Databases (SID) and the NIS, OptumInsight™ Retrospective Database (LabRx), and Premier Perspective, to improve our understanding of the burden of CDI in the U.S. from a population perspective.

## Methods

The databases used for this study were anonymized; some contained encrypted identifiers to link longitudinal data within a person (“cohort” data), while the others consisted of only unlinked hospital billing data. The hospital billing databases (NIS, Premier) were analyzed at the hospital discharge level. The cohort databases containing a person-level identifier to track persons across healthcare encounters (Medicare, LabRx, and SID) were analyzed at both the person-level and hospital discharge-level. For all cohort data hospitalizations with same-day transfers to the same or a different hospital were aggregated and treated as a single hospital stay, to avoid over-counting long hospitalizations or direct transfers as distinct hospital visits. The Washington University Human Research Protection Office and Geisel School of Medicine at Dartmouth Committee for the Protection of Human Subjects gave approval to conduct this research with a waiver of informed consent.

### Identification of CDI

Criteria used to identify CDI combined any of the following:

1. ICD-9-CM diagnosis code for CDI (008.45) during an inpatient hospital stay;
2. ICD-9-CM diagnosis code for CDI in an outpatient encounter with specific restrictions (see Additional file 1: Appendix);
3. Positive test result for *C. difficile* toxins or toxin genes (LabRx); and
4. Non-topical metronidazole or oral vancomycin therapy within ± 14 days of a CPT-4 code for a *C. difficile* test or diagnosis code for CDI (Medicare, LabRx, and Premier).

For person-level analyses, subsequent unique episodes of CDI were identified if the person met criteria for CDI again after an 84 day period during which there were no healthcare encounters meeting the CDI case definition. We used a conservative definition for subsequent unique episodes of CDI to minimize misclassifying carry-forward of the CDI diagnosis code or CDI recurrence as a unique episode of CDI.

### Inclusion/exclusion criteria

For Medicare and LabRx data, enrollment and complete health insurance coverage for the year prior to the first onset date of CDI was required. For Medicare age ≥ 66 years at the time of CDI onset was required; for the SID, NIS, and Premier all persons aged ≥ 18 years were included. Individuals 65 and older were excluded from the LabRx data since they represented only 7 % of the privately insured population. For the cohort data CDI episodes were excluded if the person had CDI within the prior 84 days in order to identify new episodes of CDI in 2009.

### Date of onset and determination of the location of onset and attribution of CDI

The date of onset of CDI was defined as the first date corresponding to a coded diagnosis of CDI. In the LabRx data, if a CDI toxin test was performed, the date of the first positive test was used as the date of CDI onset.

The location of onset and attribution for each CDI episode was determined using an algorithm based on the most recent SHEA/IDSA definitions [16–18]. CDI coded during a hospitalization was classified as community-onset if: 1) CDI was the primary diagnosis; 2) the primary diagnosis was diarrhea, abdominal pain, or nausea and CDI was coded in a secondary position; or 3) CDI was coded in a secondary position and the hospital length of stay was ≤ 3 days. If no further information was available from outpatient or physician claims, CDI was classified as hospital-onset if it was coded in a secondary position and the hospital length of stay was > 3 days. If the database did not contain a common person identifier, no further categorization beyond community- or hospital-onset was possible. If a common person identifier was available and the CDI episode was community-onset, hospitalizations and other healthcare facility exposures prior to the CDI hospital admission were identified to classify the episode (see Additional file 1: Appendix).

### Analysis

The rate of CDI in a population group was defined as the number of CDI episodes divided by the person-years of observation (pyo, defined from 1/1/2009 up to the next CDI event, death, or 12/31/2009, whichever came first). For the SID the population of adults aged 18–64 and the elderly in the seven states was obtained from the 2010 census (www.census.gov). Person-years in the SID data were calculated taking into account death (using the midpoint of the death discharge quarter to define the date of death). SAS version 9.3 and SPSS 20.0 were used for data management and analysis.

## Results

The demographic characteristics and number of hospitalizations and outpatient encounters in the different databases are shown in the Additional file 1: Appendix. In the three longitudinal datasets approximately 0.2 % of the initially identified hospitalizations coded for CDI were excluded because the patient was previously identified with CDI within the prior 12 weeks. The criteria used to identify CDI are shown in Table 1. In the Medicare data 23 % of inpatient CDI episodes were identified by the CDI diagnosis code together with an outpatient prescription for metronidazole or oral vancomycin within 14 days after hospital discharge; when restricted to patients with Part D coverage this corresponded to 40 % of inpatient CDI episodes (1303/3280). In the Medicare data approximately 53 % of unique CDI inpatient hospital episodes were identified by a secondary diagnosis code during the hospitalization, consistent with hospital-onset CDI, compared to 70 % in the SID, and 23 % in the LabRx data. In the encounter-level Premier data, 73 % of CDI hospitalizations were identified by a *C. difficile* laboratory test, diagnosis, plus metronidazole or oral vancomycin therapy, and 21 % were identified based on the CDI diagnosis code plus treatment without a positive test result.

**Table 1:** CDI Episodes in 2009 according to the definition used to identify CDI

Definition	Medicare^a^	Lab/Rx^a^	SID^a^	Premier	NIS
Positive test + diagnosis + Rx	N/A	N/A	–	4,623 (72.8)	–
Diagnosis + Rx within 14 days after hospital discharge or during hospitalization	1,303 (23.4)	1,088 (65.5)	–	1,347 (21.2)	–
Diagnosis + Positive test during hospitalization	N/A	N/A	–	108 (1.7)	–
Primary diagnosis	994 (17.8)	194 (11.7)	20,894 (30.5)	13 (0.02)	110,553 (32.8)
Diagnosis on admission	338 (6.1)	N/A	N/A	2 (< 0.01)	N/A
Positive test only	N/A	N/A	N/A	205 (3.2)	N/A
Secondary diagnosis	2,941 (52.7)	379 (22.8)	47,546 (69.5)	52 (0.08)	226,012 (67.2)
Total Inpatient CDI	5,576	1,661	68,440	6,350	336,565
Positive test + Diagnosis + Rx within +/−14 days of diagnosis or positive test date(s)	N/A	87 (2.7)	–	–	–
Diagnosis on ≥ 2 service dates + Rx within +/−14 days of diagnosis date(s)	422 (10.4)	409 (12.6)	–	–	–
Positive test + diagnosis within +/−14 days	N/A	6 (0.2)	–	–	–
Positive test + Rx within +/−14 days	N/A	436 (13.4)	–	–	–
Diagnosis + Rx + lab test within +/−14 days of the diagnosis or lab test date(s)	133 (3.3)	148 (4.6)	–	–	–
Diagnosis + Rx within +/−14 days	334 (8.2)	524 (16.1)	–	–	–
Positive test only	N/A	262 (8.1)	–	–	–
Diagnosis only	3,162 (77.6)	1,251 (38.5)	–	–	–
CDI test + Rx	25 (0.6)	129 (4.0)	–	–	–
Total Outpatient CDI	4,076	3,252	–	–	–

^a^Number of unique CDI episodes spaced at least 12 weeks apart

Rx = non-topical metronidazole or oral vancomycin therapy

Approximately 42 % of the CDI episodes in the Medicare data were first identified in the outpatient setting (Table 1). Of these outpatient CDI episodes, 78 % were identified by the CDI diagnosis code alone, and 21.8 % were identified by the diagnosis code plus outpatient CDI prescription (35 % for individuals with Part D coverage). Fifteen percent (610/4076) of the persons identified with CDI outside of the hospital were hospitalized within 14 days of CDI diagnosis; of these 60 % (364/610) were coded for CDI during the inpatient hospitalization. In the LabRx data of younger persons, 38.5 % of outpatient CDI episodes were identified by the CDI diagnosis code only with no supporting laboratory or prescription evidence for infection. A total of 28.7 % of the outpatient CDI episodes in the LabRx data were identified by a diagnosis code plus therapy, while 13.4 % of outpatient CDI episodes were identified by a positive *C. difficile* test plus therapy within 14 days.

The categorization of CDI episodes by database is shown in Table 2. Fifty-nine percent of CDI episodes (5648/9652) were categorized as healthcare facility onset (hospital or other facility) in the Medicare data, compared to 68 % in the SID (46,739/68,440). Community-onset healthcare facility-associated CDI made up 13 % of the CDI episodes in Medicare, compared to 11.8 % in the SID. Community-onset community-associated CDI episodes included 22.6 % of episodes in Medicare vs. 13.9 % in the SID and 35.2 % in the Premier data. Only 22.4 % (1102/4913) of the CDI episodes in the LabRx data were healthcare facility associated (excluding indeterminate association), while 68.4 % of episodes were categorized as community-onset community-associated.

**Table 2:** Categorization of CDI episodes in the different databases

	Medicare	Lab/Rx	SID	Premier^a^	NIS
Hospital-Onset	3,223 (33.4)	449 (9.1)	46,347 (67.7)	3,984 (64.8)	211,344 (62.8)
Other Healthcare-Facility Onset	2,425 (25.1)	54 (1.1)	392 (5.7)	–	–
Community-Onset Hospital-associated	1,258 (13.0)	599 (12.2)	8,048 (11.8)	–	–
Community-Onset Indeterminate-association	561 (5.8)	451 (9.2)	4,139 (6.0)	–	–
Community-Onset Community-associated	2,185 (22.6)	3,360 (68.4)	9,514 (13.9)	–	–
Community-Onset Unknown association^b^	–	–	–	2,161 (35.2)	125,221 (37.2)

^a^The number of CDI events is reduced by 205 due to diagnosis of CDI by a positive test result only

^b^Episodes in the Premier and NIS encounter-level databases were classified as community-onset unknown association since it was not possible to determine prior health care exposures due to lack of a person-level identifier

The number of persons with one or more than one unique episode of CDI in the longitudinal datasets is shown in Table 3 and the cumulative incidence of CDI in Table 4. 2.6 % of persons in the Medicare and 5.0 % of persons in the LabRx data had > 1 unique episode of CDI spaced at least 12 weeks apart in 2009. The rate of CDI in the Medicare data was 677/100,000 pyo, while the rate was 43 % lower (383) in the SID. The rate of CDI in the younger adult population in the SID was ten-fold lower (37.5) than the rate in the elderly SID population, while the rate of CDI in the LabRx data including outpatient CDI was 1.8-fold higher than in the SID younger population. The rate of hospital onset CDI per 10,000 patient days was higher in the SID for elderly persons (15.9) compared to the Medicare data (9.8), lower in the SID and Premier data for younger adults, and lowest (1.1) in the LabRx data.

**Table 3:** Number of persons with multiple incident CDI episodes (no other CDI diagnosis within 12 weeks)

	Medicare	LabRx	SID
Size of the cohort	1,465,927	7,255,708	5,575,935
No CDI	1,456,526	7,251,046	5,508,903
Only one CDI	9,155 (97.4)^a^	4,429 (95.0)	65,549 (97.9)
2 CDI	241 (2.6)	215 (4.6)	1,403 (2.1)
> 2 CDI	5 (0.05)	18 (0.4)	28 (0.04)

^a^Percentages refer to percentage of persons with CDI

**Table 4:** Burden of CDI in the elderly in 2009 in the different databases, including all episodes of CDI

Adults < 65 Years	Lab/Rx	SID	Premier	NIS
Rate of CDI/100,000 person-years	66.0	37.5^a^	N/A	N/A
Rate of hospital onset CDI/10,000 pt. days	1.1	5.7	5.4	6.9
Prevalence of CDI at admission/1,000 hospitalizations	N/A	1.5	1.9	2.0
Rate of healthcare facility-associated CDI/10,000 pt. days	2.1	N/A	N/A	N/A
Elderly	Medicare	SID	Premier	NIS
Rate of CDI/100,000 person-years	677	383^b^	N/A	N/A
Rate of hospital onset CDI/10,000 pt. days	9.8	15.9	11.6	15.5
Prevalence of CDI at admission/1,000 hospitalizations	5.4	4.7	6.3	6.2
Rate of healthcare facility-associated CDI/10,000 pt. days	12.5	N/A	N/A	N/A

^a^rate/100,000 persons in 7 states aged 18–64 years

^b^rate/100,000 persons in 7 states aged > = 65 years

To determine the impact of including outpatient medical claims and linkage within a person on CDI incidence, we compared the cumulative incidence, categorization of episodes, and attribution of CDI in the Medicare data when complete claims were used vs. only inpatient facility claims, with and without linkage within a person. When only the inpatient facility claims were used (analogous to the SID), the total number of CDI episodes was reduced to 6276 and the cumulative incidence of CDI decreased to 440/100,000 pyo. In addition, the number of hospital-onset cases and the rate of hospital-onset CDI increased while community-associated CDI decreased over two-fold (Table 5). When the person-level linkage in the inpatient Medicare data was removed (analogous to the NIS), the number of CDI episodes increased by almost 30 % compared to the linked inpatient Medicare data (8108 vs. 6276), because of the inability to exclude hospitalizations coded for CDI in the prior 12 weeks. The number of CDI events/100,000 hospitalizations was 553/100,000 hospitalizations using the unlinked data. When the 2009 NIS data was restricted to hospitalizations in elderly persons aged 65 years and older, the CDI hospitalization proportion was 544 CDI visits/100,000 hospitalizations.

**Table 5:** Comparison of the number of CDI episodes in 2009 in the Medicare data according to the extent of information used to identify CDI

		Source of information from medicare files		
Type of CDI	Definition of CDI	Complete inpatient + outpatient	Inpatient only (comparable to SID)	Inpatient-unlinked (comparable to NIS)
# hospital stays	–	488,344	488,344	522,921
Total Inpatient CDI episodes	–	5,576	6,276	8,108
Inpatient CDI Episodes	Primary discharge diagnosis)	1,464	1,690	2,084
	Admission diagnosis	487	584	824
	Secondary discharge diagnosis	3,625	4,002	5,200
Outpatient CDI episodes	–	4,076	N/A	N/A
Total CDI episodes	–	9,652	6,276	8,108
Categorization of CDI Episodes (% of episodes)				
Hospital-Onset		3,223 (33.4)	3,765 (60.0)	4,769 (58.8)
Other Healthcare Facility-Onset		2,425 (25.1)	N/A	N/A
Community-Onset Healthcare Facility–associated		1,258 (13.0)	938 (14.9)	N/A
Community-associated CDI		2,185 (22.6)	1,032 (16.4)	3,339 (41.2)
Indeterminate		561 (5.8)	541 (8.6)	N/A
Rate of CDI Episodes				
Rate of CDI/100,000 person-years		677	440	553^a^
Rate of hospital onset CDI/10,000 pt. days		9.8	11.4	14.1
Prevalence of CDI at admission/1,000 hospitalizations		5.4	5.3	6.5
Rate of healthcare facility-associated CDI/10,000 pt. days		12.5	14.2	14.1

^a^per 100,000 population aged 65 and older from the 2010 census

## Discussion

We used five types of billing or claims data to define the burden of CDI in U.S. adults in 2009. To our knowledge this is the first study to compare the burden of CDI from a population perspective in different administrative databases using standardized methods to identify and classify CDI. We used all available information to identify CDI, including outpatient prescription claims for metronidazole and vancomycin in the Medicare (Part D) and LabRx data, inpatient treatment in Premier, and outpatient *C. difficile* test results in LabRx.

Not surprisingly, we found a higher cumulative incidence of CDI in the databases that contained inpatient and outpatient data compared to only inpatient billing data, similar to what was reported recently using Kaiser Permanente data [19]. The number of CDI episodes per 100,000 elderly persons was almost 1.8-fold higher in the Medicare data compared to the inpatient only longitudinal-SID. However, when only inpatient data were used to identify CDI in the Medicare population and the analysis was conducted at the person-level, the cumulative incidence of CDI was very close to that calculated in the SID (440 vs. 383/100,000 pyo). The 54 % increase in the cumulative incidence using complete (677) vs. inpatient-only Medicare data (440) emphasizes the importance of using complete data from inpatient and outpatient settings to calculate CDI incidence. In addition, when we treated the inpatient Medicare data as encounter-level (i.e., hospitalizations as unique encounters), the number of CDI events/100,000 hospitalizations was remarkably similar to the 544 /100,000 hospitalizations in elderly persons in the 2009 NIS.

More CDI cases were identified as hospital-onset in the datasets with only inpatient facility data, resulting in a higher apparent hospital-onset CDI incidence. The rate of hospital-onset CDI increased in the Medicare data to 11.4 cases/10,000 patient days when analysis was restricted to only inpatient facility claims, and this rate increased further when the linkage within a patient was ignored (14.1 cases/10,000 patient days). This suggests that analysis of encounter-level data, such as the NIS, may result in over-estimation of CDI hospital rates by as much as 25 % due to continued coding in subsequent hospitalizations that are part of the same CDI episode, and that caution should be used when using these data for surveillance purposes.

In analysis of complete Medicare claims, 33 % of the CDI events were categorized as hospital-onset, whereas in the analyses using only inpatient Medicare facility data, approximately 60 % of the CDI events were categorized as hospital-onset, suggesting that hospital-onset cases will be over-estimated by almost two-fold when only inpatient claims or billing data are used. These results are consistent with previous reports of the over-attribution of hospital-onset CDI [20, 21] and the over-estimation of CDI cases identified by the ICD-9-CM diagnosis code compared to positive *C. difficile* toxin assay from facility billing data [20, 22–25]. More hospital-onset cases were identified in the HCUP and Premier data, likely due to misclassification of CDI with onset in the community.

The addition of laboratory results in the LabRx data suggests that 20 % of CDI episodes may be missed when analyzing data without *C. difficile* test results. Identification of fecal transplant in administrative data via CPT-4 and HCPCS codes (available beginning 2013) may aid in the identification of CDI in future, particularly in combination with a positive *C. difficile* test result. Interestingly, approximately three-quarters of the outpatient CDI episodes in the LabRx data were not supported by a positive test result, with 38 % identified on the basis of a CDI diagnosis alone. The percentage of outpatient CDI diagnoses without confirmation by a positive *C. difficile* laboratory test was very similar in our previous study using Veterans Administration data, in which only 32 % of the total outpatient CDI cases had a *C. difficile* test result [26]. Further studies to validate the use of the ICD-9-CM diagnosis code for CDI in the outpatient setting in the absence of positive *C. difficile* test results are warranted to determine the accuracy of coding outside of the hospital.

In the Medicare data 23 % of CDI episodes first diagnosed during an inpatient hospital stay were linked to a filled outpatient prescription consistent with CDI treatment within 2 weeks after hospital discharge. Since 47.5 % of the Medicare patients had Part D coverage, this would suggest that almost half of elderly persons diagnosed with CDI during an inpatient hospitalization continue CDI treatment after leaving the hospital. In the LabRx data almost two-thirds of episodes identified during a hospitalization were linked to outpatient CDI treatment. We identified temporally related treatment of outpatient CDI in at most one-half of persons with prescription drug coverage in the Medicare (46 %) and LabRx (49 %) data. In contrast, in the Premier data containing inpatient medications, 73 % of inpatient CDI episodes had evidence of treatment during the hospitalization. Despite lack of documentation of treatment for many CDI cases, particularly in the Medicare data, the overall incidence of CDI in the elderly of 677/100,00 pyo is remarkably similar to the incidence of 628/1000,000 elderly persons reported by the Centers for Disease Control and Prevention’s Emerging Infections Program (EIP) for 2011 [2].

Lessa reported that 53 % of CDI events (159,700 community-associated + 81,300 community-onset, health care facility associated) in persons of all ages were community-onset using the EIP data [2], similar to the 41.4 % community-onset CDI episodes we identified in the Medicare data. In the recent publication using Kaiser Permanente data, 76 % of the CDI events were community-onset, with a total of 40 % characterized as community-onset community-associated [19]. This is lower than our finding that almost 90 % of the CDI events in the LabRx data from younger persons had onset in the community, with 68 % characterized as community-onset community associated CDI. The varying proportions of CDI with onset outside of the hospital in the Medicare and LabRx data compared to the EIP data may be related to differences in age of the populations. In the EIP data, 44 % of persons with CDI were < 65 years of age, and the proportion of CDI that is community-associated CDI is higher in younger populations [27]. Consistent with our current study, 52 % of laboratory-identified CDI during inpatient hospitalizations in the EIP were present at admission to the hospital [28].

## Conclusions

The similarities between our findings concerning the incidence and site of onset of CDI from several different administrative databases with recent EIP results validate use of these administrative databases to identify populations at risk for CDI. We determined how results may be skewed when important information is missing, such as outpatient data and encrypted identifiers, and the advantages of using complete claims data allowing for substantiation of the CDI diagnosis using laboratory claims for CDI testing, pharmacy claims for CDI treatment, and other diagnoses consistent with CDI (e.g., diarrhea). Although there are limitations to use of administrative data, these databases offer the opportunity to analyze CDI from a population perspective, including data from many different hospitals and from other healthcare facilities. These databases can provide more complete information on the epidemiology of CDI and enrich our understanding of the impact of CDI on young and older persons in the U.S. In addition, the methods we developed to extract comparable information can be used to determine the incidence of other infectious diseases (e.g., MRSA, septicemia) and adverse events (e.g., deep venous thrombosis) in varying populations using a combination of different administrative databases.

## Ethics approval and consent to participate

The Washington University Human Research Protection Office and Geisel School of Medicine at Dartmouth Committee for the Protection of Human Subjects gave approval to conduct this research with a waiver of informed consent.

## Consent for publication

Not Applicable.

## Availability of data and materials

Five different data sources were used for this study. None of the data sources can be shared by the authors, per data use agreements with the individual organizations. All of the data sources are available for purchase, as described below.

Medicare claims (Chronic Condition Warehouse 5 % random sample), obtained from the Centers for Medicare and Medicaid Services and the Research Data Assistance Center (www.resdac.org).

OptumInsight™ Retrospective Database (formerly i3 Ingenix/LabRx), obtained from OptumInsight (www.optum.com).

Healthcare Cost and Utilization Project Nationwide Inpatient Sample (NIS) and State Inpatient Databases (SID), obtained from the Agency for Healthcare Research and Quality (www.hcup-us.ahrq.gov/databases.jsp).

Premier Perspective database, obtained from Premier, Inc (www.premierinc.com).

## Additional file

Additional file 1: Appendix. Description of Databases. Appendix 1 includes descriptions of the cohort and hospital billing databases used in the study. Appendix 2. Definition and characterization of *Clostridium difficile* infection. Appendix 2 includes study inclusion and exclusion criteria and identification and classification of CDI. Appendix 3. Information used from Different Databases to Identify CDI. Table includes information available in the different databases that was used to identify CDI. Appendix 4. Comparison of Number of Persons and Encounters in the Different Databases. Table includes the initial number of persons, hospitalizations, and outpatient visits identified in the five databases and the final numbers after applying exclusion criteria. Appendix 5. Demographics of Populations from the Different Databases. Table includes the demographics of persons or hospital encounters in the five databases. (DOCX 25 kb)

## Abbreviations

CCW: Chronic Condition Warehouse
CDI: *C*lostridium difficile infection
CMS: Centers for Medicare and Medicaid Services
CPT-4: Current Procedural Terminology, 4^th^ edition
EIP: Emerging Infections Program
HCUP: Healthcare Cost and Utilization Project
ICD-9-CM: International Classification of Diseases, Ninth Revision, Clinical Modification
LabRx: OptumInsight LabRx data
MRSA: methicillin-resistant *S*taphylococcus aureus
NIS: Nationwide Inpatient Sample
pyo: person-years of observation
SID: State Inpatient Database
