# Supplementary material for: The burden of clostridium difficile infection: estimates of the incidence of CDI from U.S. Administrative databases
Source: BMC Infect Dis. 2016 Apr 22;16:177. doi: 10.1186/s12879-016-1501-7 (PMC4840985; doi:10.1186/s12879-016-1501-7)
Supplement: Additional file 1: Appendix. — Description of Databases. Appendix 1 includes descriptions of the cohort and hospital billing databases used in the study. Appendix 2. Definition and characterization of Clostridium difficile infection. Appendix 2 includes study inclusion and exclusion criteria and identification and classification of CDI. Appendix 3. Information used from Different Databases to Identify CDI. Table includes information available in the different databases that was used to identify CDI. Appendix 4. Comparison of Number of Persons and Encounters in the Different Databases. Table includes the initial number of persons, hospitalizations, and outpatient visits identified in the five databases and the final numbers after applying exclusion criteria. Appendix 5. Demographics of Populations from the Different Databases. Table includes the demographics of persons or hospital encounters in the five databases. (DOCX 25 kb) [file 12879_2016_1501_MOESM1_ESM.docx]

**Additional file 1 (Appendix 1-5)**

**Appendix 1. Description of Databases**

**Cohort Databases**

The Centers for Medicare and Medicaid Services (CMS) Chronic Conditions Data Warehouse (CCW) 5% sample database was used for the Medicare analyses. The files used to identify patients with CDI included the Inpatient, Carrier Claims (physicians and other medical services), Outpatient (outpatient facility services), Beneficiary Summary, Skilled Nursing Facility, and Part D Drug Event. The files contain claims data with ICD9-CM diagnosis and procedure codes, Healthcare Common Procedure Coding System (HCPCS)/Current Procedural Terminology (CPT^®^) codes, dates of service, line item detail, reimbursement amount, and beneficiary Medicare enrollment and demographic information. The Part D drug event file contains the National Drug Code (NDC) to identify outpatient drug utilization, quantity dispensed, days supplied, route and dosage, and payment information. Each observation in these files is at the claim level, but can be combined to the person level using the encrypted beneficiary ID. Individuals were excluded if they were enrolled at any time in 2008-2009 in a health maintenance organization or lacked Part A or Part B coverage which would result in incomplete claims data.

The OptumInsight™ Retrospective Database (formerly i3 Ingenix/LabRx) contains data for a sample of employed individuals insured by the commercial insurer United Healthcare. The OptumInsight LabRx data includes inpatient and outpatient medical claims, outpatient prescription drug claims, and outpatient laboratory results for persons in a variety of different health plans.

**Hospital Billing Databases**

The Premier Perspective database (Premier Inc., Charlotte, NC) contains clinical and billing data from hospitals participating in Premier’s healthcare alliance program. Perspective is a voluntary, fee-based program with over 2,500 participating U.S. hospitals (including academic medical centers, community-based hospitals, and large multihospital systems). The Premier database contains standard hospital discharge data (ICD-9-CM diagnosis and procedure codes, revenue codes) at the person-level, in addition to detailed daily billed services utilization, including medications, laboratory tests, diagnostic and therapeutic services.

The HCUP data used in this study included 7 State Inpatient Databases (SID) and the Nationwide Inpatient Sample (NIS). The SID databases for a number of participating states are available through HCUP, and include all inpatient hospital discharge records from non-federal/state community hospitals that submitted data to their state data organization. The NIS is a stratified sample of hospitals from the SID databases (numbering 46 states as of 2011), and contains the discharge records from about 1000 hospitals per year, approximately a 20% stratified sample of hospitals in the U.S. The SID and NIS contain information on demographics (e.g., age, gender, median community-level income and in some cases race/ethnicity, ICD-9-CM diagnosis and procedure codes, diagnoses present-on-admission, expected payer, admission source, and discharge disposition. The NIS database can only be analyzed at the discharge encounter level, since no patient-level identifiers are present. Some of the individual SID databases include an encrypted patient-level identifier variable, and thus analyses of inpatient hospital discharges can be performed at the patient-level for these states. For this study seven states with the encrypted patient-level identifier that spanned 2008-2009 were used (Arkansas, California, Florida, Hawaii, Nebraska, New York, and North Carolina). The American Hospital Association annual survey data were used to exclude non-community hospitals in the SID (i.e., federal/state, psychiatric, rehabilitation, alcohol/chemical dependency, and long-term care facilities), since inclusion of data from those facilities is variable across states.

**Appendix 2. Definition and characterization of *Clostridium difficile* infection.**

**Identification of CDI**

Common definitions were applied to all databases to identify CDI depending on whether the database contained cohort information or just inpatient billing data. Criteria used to identify CDI combined any of the following:

1) ICD-9-CM diagnosis code for CDI (008.45) during an inpatient hospital stay;

2) ICD-9-CM diagnosis code for CDI in an outpatient encounter (excluding diagnosis codes on claims with a place of service code of laboratory, provider type code of laboratory, or type service code of diagnostic laboratory, or if the only revenue center codes on the facility claim were for laboratory (UB-92 revenue codes 0300-0309));

3) Positive test result for *C. difficile* toxins or toxin genes (LabRx only); and

4) Non-topical metronidazole or oral vancomycin therapy within ± 14 days of a CPT-4 code for a *C. difficile* test (87230, 87324, 87449, 87803, in the absence of known test results) or within ± 14 days of a 008.45 diagnosis code for CDI).

All pertinent data available within a database were used to identify an individual’s first CDI case in 2009. For person-level analyses, subsequent unique episodes of CDI within a person were identified if the person met criteria for CDI again after an 84 day (12 weeks) period during which there were no healthcare encounters in which the person met the case definition for CDI. This conservative definition to identify subsequent unique episodes of CDI was used to minimize misclassifying carry forward of the ICD-9-CM code for CDI or a recurrent episode of CDI as a unique episode of CDI within a person.

**Additional Inclusion/Exclusion Criteria**

In addition to the inclusion/exclusion criteria described in the text, for all databases individuals or encounters were excluded if they were missing information on age. For the SID databases, encounters with missing encrypted patient-level identifier were excluded since it would not be possible to link hospital discharges within a person. For the cohort data CDI episodes were excluded if the person had CDI (as defined in Table 1) within the prior 84 days (including data from the last quarter of 2008) in order to identify new episodes of CDI in 2009. We used a conservative window of 84 days to identify incident infections in order to avoid over-counting episodes in the claims data due to repeated coding of CDI during follow-up health care encounters.

**Date of Onset of CDI**

The date of onset of CDI was defined as the first date corresponding to a coded diagnosis of CDI, unless additional information was available to define an earlier date as the date of onset (See Appendix). The additional information used to define an earlier date of symptom onset was a diagnosis of diarrhea (ICD-9-CM diagnosis codes 009.0-009.3, 787.91), abdominal pain (789.00-789.09) or nausea (787.01, 787.02), or CPT-4 codes in outpatient files for stool microbiology tests (87045, 87046, 87177, 87269, 87329, 87272, 87328, 87335, 87427, 87425). If any of these were present, the date of onset was the earliest date of the CDI indicator within the 28 days before the CDI diagnosis code. In the databases with laboratory results (LabRx), if a CDI toxin test was performed, the date of the first positive test was used as the date of CDI onset.

**Date of Onset and Determination of the Location of Onset and Attribution of CDI**

The date of onset of CDI was defined as the first date corresponding to a coded diagnosis of CDI, In the LabRx data, if a CDI toxin test was performed, the date of the first positive test was used as the date of CDI onset.

Determination of the location of onset and attribution for each CDI episode was done using an algorithm based on the most recent SHEA/IDSA guideline definitions. CDI coded during a hospitalization was classified as community-onset if: 1) CDI was the primary diagnosis; 2) the primary diagnosis was diarrhea, abdominal pain, or nausea and CDI was coded in a secondary position; or 3) CDI was coded in a secondary position and the hospital length of stay was ≤ 3 days. If no further information was available from outpatient or physician claims (i.e., Premier, SID, NIS), CDI was classified as hospital-onset if it was coded in a secondary position and the hospital length of stay was > 3 days. If the database did not contain a common person identifier, no further categorization beyond community- or hospital-onset was possible.

For all datasets, if CDI was first coded during a hospitalization by the facility in a secondary position, the primary diagnosis was not diarrhea, abdominal pain, or nausea, and the length of stay was > 3 days, the CDI episode was categorized as hospital-onset. For the Medicare and LabRx databases with provider claims, that information was used to help determine the onset and attribution of CDI. If the date of the first provider claim coded for CDI was nested in an inpatient hospitalization (≥ hospital admission date and ≤ hospital discharge date), that date was used as the date of CDI onset. If the provider claim for CDI was dated ≤ 3 days after hospital admission, the person was not a resident in a long term care facility (see below) and did not have any hospitalizations in the 84 days prior to admission, the CDI episode was categorized as community-onset, community-associated CDI. If the first provider claim coded for CDI was dated > 3 days after the hospital admission date, the episode was categorized as hospital-onset CDI with date of onset equal to the date of the first provider claim coded for CDI. If there were no claims information available to determine the date of hospital-onset CDI, the midpoint between hospital day 3 and discharge was considered the date of onset.

If a common person identifier was available and the CDI episode was classified as community-onset, hospitalizations prior to the CDI hospital admission were identified. If the person had been hospitalized with a discharge date ≤ 28 days before the CDI admission date, the CDI episode was classified as community-onset, healthcare facility-associated. If the person had been hospitalized with a discharge date > 28 days and ≤ 84 days before the CDI admission date, the CDI episode was classified as community-onset, indeterminate association. If the person had evidence for residence in a skilled nursing or long-term care facility (see below), the same cutoffs for designating healthcare facility or indeterminate association were used. If the person did not have any hospitalizations or stays in a skilled nursing facility, and was not identified as a nursing home resident in the 84 days prior to the CDI admission, the CDI case was categorized as community onset, community associated CDI.

We used the validated algorithm of Yun et al. [1] to identify residents of long-term care facilities in the Medicare data. In this algorithm Medicare beneficiaries are classified as residents of long term care facilities if they had claims with CPT-4 codes for physician visits to a nursing facility or nursing facility place of service codes. Entry into a long term care facility was defined as the first month in which nursing facility service claims were identified, in the absence of claims from a skilled nursing facility. For community-onset CDI, if the person met criteria for residence in a long term care facility or a skilled nursing facility the CDI episode was categorized as other healthcare facility-onset.

1. Yun H, Kilgore ML, Curtis JR, et al. Identifying types of nursing facility stays using medicare claims data: an algorithm and validation. Health Serv Outcomes Res Method 2010;10:100-10.

**Appendix 3. Information used from Different Databases to Identify CDI**

|  | **Data Source** | | | |
| --- | --- | --- | --- | --- |
| **Criteria for CDI** | **Medicare** | **LabRx** | **Premier** | **SID/NIS** |
| Inpatient facility 008.45 diagnosis code | X | X | x | X |
| Inpatient facility therapy |  |  | x |  |
| Outpatient facility or provider 008.45 diagnosis code (excluding laboratory claims) | X | X |  |  |
| Outpatient CDI therapy + (CPT-4 code for CDI test OR claim with a 008.45 diagnosis code) | X | X |  |  |
| Outpatient CDI test results |  | X |  |  |

**Appendix 4. Comparison of Number of Persons and Encounters in the Different Databases**

| **Characteristic** | **Medicare** | **LabRx** | **SID** | **Premier** | **NIS** |
| --- | --- | --- | --- | --- | --- |
| Population size | 1,465,927 | 7,255,708 | 69,541,350* | N/A | 234,564,071* |
| Number of persons with at least one hospitalization | 279,797 | 467,693 | 5,575,935 |  |  |
| Total number of hospitalizations | 522,921 | 611,663 | 8,082,776 | 928,214 | 33,094,451 |
| Excluded transfer hospitalizations (% of total hospitalizations) | 34,577 (6.61%) | 21,823 (3.57%) | 204,494 (2.53%) |  |  |
| Excluded hospitalizations due to CDI hospitalization in prior 12 weeks (% of total hospitalizations) | 1,215 (0.23%) | 1,353 (0.22%) | 14,817 (0.18%) |  |  |
| Final number of hospitalizations with all exclusions (same-day transfers + CDI in the prior 12 weeks) | 488,344 | 588,608 | 7,863,465 |  |  |
| **Outpatient Information** | | | | | |
| Number of persons with at least one outpatient encounter | 1,297,518 | 7,255,038 |  | | |
| Number of outpatient visits | 11,385,402 | 60,748,363 |  | | |

*****based on 2010 census data

| **Cohort Databases – Number of Persons** | | | |
| --- | --- | --- | --- |
| **Characteristic**  **N (%)** | **Medicare**  **(n=1,465,927)** | **LabRx**  **(n=7,255,708)** | **SID**  **(n=5,575,935)** |
| Age  18-64  65+ | 0  1,465,927 (100%) | 7,255,708 (100%)  0 | 3,433,863 (61.6%)  2,142,072 (38.4%) |
| Female | 897,227 (61.2%) | 4,064,815 (56.02%) | 3,450,338 (61.9%) |
| Race  White  Black  Hispanic  Other/Missing | 1,266,929 (86.4%) |  | 3,000,249 (60.2%)**  617,839 (12.4%)  767,261 (15.4%)  602,224 (12.1%) |
| Health insurance  Medicare primary  Medicaid  Private  Uninsured/other | 1,465,927 (100%)  186,144 (12.7%)*  N/A  0 | 186 (0.003%)  143,756 (2.0%)  7,111,766 (98.0%)  0 | 2,224,254 (39.9%)  943,618 (16.9%)  1,867,474 (33.5%)  540,589 (9.7%) |
| **Billing Databases – Number of Encounters** | | | |
| **Characteristic**  **N (%)** | **Premier**  **(n=928,214)** | **NIS**  **(n= 33,094,451)** |  |
| Age  18-64  65+ | 544,573 (58.7%)  383,641 (41.3%) | 19,550,279 (59.1%)  13,544,172 (40.9%) |  |
| Female | 568,403 (61.2%) | 19,814,451 (59.9%) |  |
| Race  White  Black  Other | 631,917 (68.1%)  158,294 (17.1%)  138,003 (14.9%) | 22,738,298 (68.7%)  4,544,674 (13.7%)  5,811,480 (17.6%) |  |
| Health insurance  Medicare primary  Medicaid  Private  Uninsured/other | 400,158 (43.1%)  126,908 (13.7%)  341,672 (36.8%)  59,476 (6.4%) | 14,687,526 (44.4%)  5,048,648 (15.3%)  12,195,594 (36.9%)  1,162,683 (3.5%) |  |

**Appendix 5. Demographics of Populations from the Different Databases**

*Medicaid dual coverage

******race in SID-longitudinal excluding NC and NE (race not reported)
